# Initial investigation of dietitian perception of plant-based protein quality

**DOI:** 10.1002/fsn3.112

**Published:** 2014-04-29

**Authors:** Glenna J Hughes, Kathleen S Kress, Eric S Armbrecht, Ratna Mukherjea, Mildred Mattfeldt-Beman

**Affiliations:** 1 Department of Nutrition and Dietetics, Saint Louis UniversitySt. Louis, Missouri; 2 Global Nutrition, DuPont Nutrition & HealthSt. Louis, Missouri; 3 Center for Outcomes Research (SLUCOR), Saint Louis UniversitySt. Louis, Missouri

**Keywords:** Beliefs and knowledge, dietitian, plant-based protein, protein quality

## Abstract

Interest in plant-based diets is increasing, evidenced by scientific and regulatory recommendations, including Dietary Guidelines for Americans. Dietitians provide guidance in dietary protein selection but little is known about how familiar dietitians are with the quality of plant versus animal proteins or methods for measuring protein quality. Likewise, there is a need to explore their beliefs related to dietary recommendations. The aim of this study was to assess dietitians' perceptions of plant-based protein quality and to determine if these are affected by demographic factors such as age and dietary practice group (DPG) membership. This was a cross-sectional design using an online survey. The survey was sent to all members of the Missouri Dietetic Association. All completed surveys (136) were analyzed. The main outcome measures were responses to belief and knowledge questions about the protein quality of plant-based diets, along with demographic information including age and DPG membership. Descriptive statistics and frequencies were determined, and chi-square analysis was used to determine the associations between belief and knowledge responses and demographic characteristics. Responses to belief statements suggested a high level of support for plant-based diets. No associations were found between any of the belief questions and demographic factors. A majority of respondents were not familiar with protein quality determination methods that are currently recognized by global regulatory and advisory agencies. Potential barriers identified in shifting to a more plant-based diet were lack of interest and perceived difficulty. Knowledge among dietitians of plant-based protein quality in general, and methods of protein quality measurement more specifically, needs to be addressed to enhance their knowledge base for making dietary protein recommendations. Two potential avenues for training are university curricula and continuing education opportunities provided to practitioners who provide dietary advice.

## Dietitian Perception of Plant-Based Protein Quality

Interest in plant-based diets is increasing, evidenced by recommendations by scientific and regulatory bodies for increased consumption of plant-based foods. The 2005 Dietary Guidelines for Americans called for increased intakes of fruits and vegetables (US Department of Agriculture and US Department of Health and Human Services 2010a). This recommendation was reinforced and expanded upon in the Report of the Dietary Guidelines Advisory Committee on the Dietary Guidelines for Americans, 2010, which advised a shift in food intake patterns to a more plant-based diet that emphasizes vegetables, cooked dry beans and peas, fruits, whole grains, nuts, and seeds; although the dry bean recommendation was cut in half from 2010 to 2005 (US Department of Agriculture and US Department of Health and Human Services 2010b). Likewise, the American Institute for Cancer Research recommends that individuals increase consumption of fruits and vegetables in their diet (World Cancer Research Fund/American Institute for Cancer Research 2007). Many health benefits have been attributed to plant-based diets, particularly related to the prevention of chronic diseases such as heart disease (Ferdowsian and Barnard 2009; Jenkins et al. 2009; Chainani-Wu et al. 2011), type 2 diabetes (Barnard et al. 2009; Salas-Salvado et al. 2011), certain cancers (Lanou and Svenson 2010), and overall mortality (Orlich et al. 2013).

As a result of such research findings and advisory recommendations, it might be expected that the number of Americans adopting vegetarian diets would be increasing. The Vegetarian Resource Group, in polls conducted in 2006, 2009, and 2011, reported the number of adults who say they never eat meat, poultry, and fish/seafood (vegetarian) at 2.3%, 3%, and 5%, respectively (The Vegetarian Resource Group 2011a,b, 2012). Due to possible sources of error, they state they do not have enough information to conclude that the number of vegetarians is changing but it is at least holding steady (The Vegetarian Resource Group 2012). However, the number remains relatively small. Semi-vegetarians or flexitarians are estimated at 13–14%, based on a poll conducted in 2005 (The Vegetarian Resource Group 2013). According to consumption statistics prepared by the American Meat Institute (American Meat Institute 2011) using USDA data, while beef consumption has trended downward over the past two decades, it has remained at an average of 66 pounds per person over the past 10 years. Pork has remained stable at 55 pounds per person over the past two decades, with red meat representing 55% of all meat (red meat, poultry, and fish) consumed. Clearly, much remains to be done to shift Americans toward a more plant-based diet.

One consequence of such a shift in dietary patterns would be the replacement of a greater portion of dietary animal protein with plant protein. Animal proteins are generally considered to be of higher quality than plant proteins, and are referred to as complete proteins. Most plant proteins, while still containing all of the essential amino acids, provide insufficient levels of one or more essential amino acid relative to biological needs, and are termed incomplete proteins. They may also be of lower digestibility than animal proteins. An exception is soy protein isolate, a plant protein that is recognized as a complete protein, with high digestibility, comparable to milk, meat, and eggs in its ability to meet human protein needs (Young 1991; Craig and Mangels 2009). The concept of consuming complementary proteins is generally recognized as a means for the general population of vegetarians to meet their protein needs (Craig and Mangels 2009).

The quality of a protein depends on its ability to provide amino acids in adequate amounts to meet the requirements of humans. Over the years, a number of methods have been employed for assessing protein quality. The protein efficiency ratio (PER) method has been in use since 1919 and was recognized in the United States for many years as the standard method for protein quality evaluation. PER is a rat assay method, based on the ability of a protein to support growth in young, rapidly growing rats. Proponents of plant protein-based diets believed that the PER method overestimated the value of some animal proteins for human growth while underestimating the value of some plant proteins, since rodents have a high sulfur amino acid requirement relative to human requirements and sulfur amino acids are the most limiting for many plant proteins (FAO/WHO 1991). PER is no longer widely used by most global regulatory bodies.

The Codex Committee on Vegetable Proteins recognized the need for a suitable indicator to express the quality of vegetable proteins and a new method was developed in the 1970s and 1980s. As information on human amino acid requirements became available, the Codex Committee concluded that a method based on comparing the amino acid composition of a dietary protein to a reference amino acid profile reflecting human needs, adjusting for digestibility, was the most suitable method for assessing protein quality for human nutrition. This work culminated in the issuance of the Joint FAO/WHO Expert Consultation on Protein Quality Evaluation (FAO/WHO 1991), in which the protein digestibility-corrected amino acid score (PDCAAS) was described and recommended for adoption. This method has become the standard method for protein quality evaluation in the United States, with the Institute of Medicine referencing its use in the development of Dietary Reference Intakes for proteins (Institute of Medicine of the National Academies 2005) and the FDA adopting the PDCAAS method for nutrition labeling of proteins in foods since 1993 and continuing to the present (FDA 2010). The Academy of Nutrition and Dietetics (AND) makes reference to PDCAAS as the standard method for determining protein quality, in its position paper on vegetarian diets (Craig and Mangels 2009). The amino acid score (AAS) is a subset of the PDCAAS method, taking the amino acid composition into account but not adjusting for digestibility.

Another method of protein quality evaluation, biological value (BV), uses nitrogen balance as a key determinant of protein quality (Hoffman and Falvo 2004). This method has been criticized for not accounting for factors such as protein modification before absorption, and for testing animals in the fasted state, among other things (Hoffman and Falvo 2004). Although a popular method among athletes, BV is not recognized by regulatory agencies for the assessment of protein quality. More recently, an FAO expert consultation proposed a new method titled Digestible Indispensable Amino Acid Score, which needs further evaluation. PDCAAS continues to be the globally recognized protein quality methodology.

Dietitians routinely assess the protein needs of their patient or client as part of the nutritional assessment process. They also counsel their clients on dietary choices and may find themselves needing to make judgments based on the quality of the protein in the diet, particularly if their clients are vegetarian, or are interested in shifting to a more plant-based diet. They may also find themselves addressing client's questions about the relative quality of different proteins, as many claims for the superiority of various proteins are found in the popular press and on the internet. As they interact with their patients and clients, dietitians have the opportunity to provide guidance on the selection of protein sources. Little is known, however, about how familiar dietitians are with the relative quality of different dietary proteins; with methods for measuring protein quality; and what their beliefs are toward the importance of such knowledge in making dietary recommendations. The objective of this study was to assess beliefs and knowledge among dietitians about the quality of protein in plant-based diets and to determine if these are affected by demographic factors such as age, education level, year degree was received, practice setting, and dietary practice group (DPG) affiliation.

## Methods

### Study design and sample recruitment

The study was conducted as a cross-sectional exploratory survey, using an online service (SurveyMonkey). Participants were recruited from the membership of the Missouri Dietetic Association (MDA), which has 1315 members. This study was approved by the Saint Louis University Institutional Review Board.

### Questionnaire development

Since this was a new area of inquiry, the questionnaire had to be developed. The survey included demographic data that might be correlated with the belief and knowledge questions. Demographics included age, gender, highest degree attained in nutrition and year obtained, registered dietitian status, zip code of practice, practice setting, number of meetings/conferences attended, and DPG membership. A question to assess client interest in dietary protein change and a question to assess where information on protein quality was obtained were also included. Six belief statements, to assess dietitians' perceptions about the relative quality of animal versus plant protein as well as the importance of such knowledge to their practice, were developed, using a 5-point Likert scale (strongly agree to strongly disagree). These were followed by five knowledge questions, with multiple choice responses, to assess familiarity with the protein quality of various plant proteins and with methods for protein quality measurement. Three final questions addressed barriers to implementation of a more plant-based diet, both from the dietitian's and the client's perspective. To establish content validity, all questions were reviewed by protein and nutrition experts for factual content. Face validity was established by pretesting the survey with a group of 17 dietetic interns to determine if any questions were confusing and to assess the length of time needed to complete the survey. The final questionnaire was reviewed by the board of the MDA prior to granting approval to survey their members.

### Data analysis

Data were analyzed using SPSS software (version 19; IBM, Armonk, NY). Descriptive statistics were used to characterize the appropriate demographics of the sample and frequencies were determined for all appropriate questions. Chi-square and *t*-test were used to compare the sample to the MDA membership in terms of age, gender, and DPG membership. Chi-square analysis was used to look at associations between belief and knowledge responses and demographic characteristics. For the purpose of this analysis, age was grouped as younger respondents (<mean age) and older respondents (≥mean age); DPG membership was grouped as members of a DPG versus nonmembers; type of degree in nutrition was grouped as undergraduate versus graduate; and year degree was received was grouped as received before 1993 versus received in 1993 or later (1993 was the year in which the PDCAAS method was first recognized by the FDA). Belief responses were collapsed into three categories: strongly agree/agree (A), neither agree or disagree (N), and disagree/strongly disagree (D).

## Results

There were 136 complete surveys used in the data analyses. When the demographics of the study sample were compared to information available for the overall MDA membership, the mean age of the sample (42 years) was older than the MDA membership (40 years) but this difference was not significant (*P* = 0.20). The sample was predominantly female and not significantly different from MDA membership (98.5% vs. 97.1%, respectively, *P* = 0.94). The sample included 61 study participants (45%) who were not a member of any DPG; this is comparable to the MDA membership (53%; *P* = 0.08). Selected characteristics of the 136 survey completers are summarized in Table 1. There was a diversity of practice settings, using the AND-defined categories, with 18 of 22 represented. Membership in DPGs was also quite diverse with 22 of the 28 DPGs represented; only one respondent indicated membership in the Vegetarian Nutrition group. In response to the question of percent of clients expressing an interest in replacing animal protein with plant protein, results indicated that this is quite low, with 46% responding that 5% or less of their clients expressed such an interest. Respondents reported using a wide range of sources for information on protein quality, with only 11% responding that they do not look for such information.

**Table 1:** Selected characteristics of survey respondents (*n* = 136)

Characteristic	Frequency (%)
Age (years)^1^	41.9 (22–78)
Gender	
Female	134 (98.5)
Male	2 (1.5)
Highest degree in nutrition	
Bachelors	78 (57.5)
Masters	40 (29.4)
Ph.D.	4 (2.9)
Other	14 (10.3)
Registered dietitian	124 (91.2)
DPG membership	
Not a member of a dietetic practice group	61 (44.9)
Vegetarian nutrition	1 (0.7)
Practice setting (most frequent)	
None indicated	25 (18.4)
Clinical nutrition (general)	24 (17.6)
Education	15 (11.0)
Number of professional meetings/conferences attended within last 5 years	
None	3 (2.2)
1–2	33 (24.3)
3–5	58 (42.6)
6 or more	42 (30.9)
Percent of clients who expressed an interest in replacing animal protein with plant protein (past 6 months)	
None	35 (25.7)
1–5	28 (20.6)
6–10	10 (7.4)
11–20	2 (1.5)
>20	4 (2.9)
Source(s) of information on protein quality^2^	
College training	78 (57.4)
ADA communications	67 (49.3)
Scientific journals	62 (45.6)
Government agencies (CDC, USDA, FDA, HHS, etc.)	59 (43.4)
Professional meetings and conferences	57 (41.9)
Internet	57 (41.9)
Continuing education classes	36 (26.5)
Do not look for information on protein quality	15 (11.0)

1 *n* = 133, 3 missing values. Values are mean (range).

2 More than one answer permitted.

The majority of respondents disagreed/strongly disagreed with the statements “the only high quality proteins are animal proteins” (D = 77.2%, N = 12.5%, A = 10.3%) and “all plant proteins are incomplete” (D = 65.4%, A = 22.1%, N = 12.5%). A high percent agreed/strongly agreed with the statement about combining plant proteins to form complete proteins (A = 93.4%, D = 4.4%, N = 2.2%). Regarding whether they would have concerns recommending a shift to more plant-based diet due to concerns about consuming adequate essential amino acids, the majority indicated they would not (D = 65.4%, A = 18.4%, N = 16.2%). However, on the question of how important an understanding of differences in protein quality between animal versus plant proteins was to their practice, respondents were more divided, with 41.9% indicating they agreed/strongly agreed versus 27.9% disagreed/strongly disagreed. Finally, a majority of the respondents indicated that they take into account the relative quality of dietary proteins when making diet recommendations (A = 64.7%, N = 25%, D = 10.3%).

**Figure 1:**
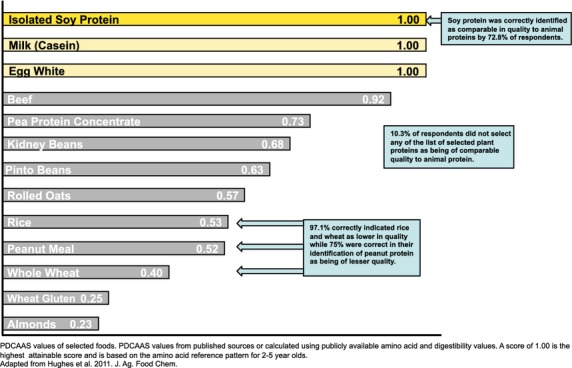
Assessments of respondents' general knowledge of protein quality of common proteins.

Two survey questions addressed general knowledge of plant protein quality. In response to the question about the quality of particular plant proteins compared to animal proteins, 97.1% correctly indicated rice and wheat as lower in quality while 75% were correct in their identification of peanut protein as being of lesser quality. Soy protein was correctly identified as comparable in quality to animal proteins by 72.8% of respondents while 10.3% did not select any of these plant proteins as being of comparable quality to animal protein. Figure 1 illustrates how respondents' knowledge of protein quality values compared to published protein quality values (Hughes et al. 2011). The second general question addressed what criteria should be included in determining the protein quality of a food. For the three correct responses, amino acid composition of the food was selected most frequently (79.4%), followed by digestibility of the food protein (64%) and amino acid needs of humans (59.6%). The majority of respondents (83.1%) correctly indicated that whether the food was plant or animal-based was not a criteria.

The other three knowledge questions were directed at specific methods of protein quality measurement. The first of these assessed familiarity with different methods of protein quality measurement, with the highest percent associated with BV (77.9%), while PER, PDCAAS, and AAS were much lower (23.5%, 18.4%, and 16.9%, respectively), and 15.4% were not familiar with any of these methods. The next question asked which of the methods is used by FDA for nutrition labeling with only 10.3% of respondents correctly selecting PDCAAS. Similarly, when asked which method is cited by AND in their position paper on vegetarian diets, only 11% were able to identify PDCAAS as the method.

The final questions of the survey addressed barriers to shifting to a more plant-based diet, and the results are summarized in Table 2. A high percentage of respondents (75%) indicated that they personally were trying to make the shift. The barriers most frequently cited, both for the dietitians personally and for their clients, were lack of interest and perceived difficulty.

**Table 2:** Responses to questions about barriers to shift to a more plant-based diet

Question	Frequency (%)of response
Recent recommendations call for a shift in food intake patterns to a more plant-based diet. As a dietitian, have you personally tried to move to a more plant-based diet?	
Yes	102 (75)
No	34 (25)
If no, what are some barriers to you implementing this recommendation for yourself?^1^	
Lack of interest in consuming plant proteins	21 (15.4)
Perceived difficulty in following a plant-based diet	12 (8.8)
Lack of knowledge about the quality of plant proteins	7 (5.1)
Lack of knowledge about the health benefits of consumingplant proteins	5 (3.7)
Other	10 (7.4)
Reflecting on the clients that you work with, what are some barriers to implementing this recommendation?^2^	
Lack of interest in consuming plant proteins	91 (66.9)
Perceived difficulty in following a plant-based diet	79 (58.1)
Lack of knowledge about the quality of plant proteins	63 (46.3)
Lack of knowledge about the health benefits of consumingplant proteins	51 (37.5)
Other	28 (20.6)

1 More than one answer permitted, *n* = 34.

2 More than one answer permitted, *n* = 136.

No associations were found between any of the belief responses and demographic factors or knowledge responses. Associations with age and DPG membership were found with responses to two of the knowledge questions (see Table 3). A significant association was found between age and one of the responses to the knowledge question regarding which plant proteins meet dietary protein needs as effectively as animal protein, with older respondents being more likely to select peanut protein (an incorrect response) than younger respondents (*P* = 0.038). Associations with age and DPG membership were also found with some of the responses to the knowledge question addressing familiarity with protein quality methods. Younger respondents were more likely to indicate familiarity with BV versus older respondents (*P* = 0.031) while older respondents were more likely to indicate that they were not familiar with any of the protein quality methods (*P* = 0.027). Respondents who were members of a DPG were more likely to indicate familiarity with the AAS method than nonmembers (*P* = 0.043). No other associations were identified between any knowledge questions and demographic characteristics.

**Table 3:** Associations between age and DPG membership and responses to knowledge questions

Question	Frequency answering YES	Frequency answering YES	*P*-value^1^
Which of the following plant-based proteins when used by themselves can meet dietary protein needs as effectively as animal-based proteins? (select all that apply)	Age group <42 years	Age group >42 years	
Peanut protein	12	22	0.038
Rice protein	3	1	*
Soy protein	51	48	ns
Wheat protein	3	1	*
None of the above	7	7	ns
I don't know	9	8	ns
Which of the following methods of protein quality measurement are you familiar with? (select all that apply)	Age group <42 years	Age group >42 years	
Biological value (BV)	59	47	0.031
Protein efficiency ratio (PER)	13	19	ns
Protein digestibility-corrected amino acid score (PDCAAS)	14	11	ns
Amino acid score (AAS)	11	12	ns
None of the above	6	15	0.027
Which of the following methods of protein quality measurement are you familiar with? (select all that apply)	DPG member	DPG nonmember	
Biological value (BV)	45	61	ns
Protein efficiency ratio (PER)	14	18	ns
Protein digestibility-corrected amino acid score (PDCAAS)	9	16	ns
Amino acid score (AAS)	6	17	0.043
None of the above	11	9	ns

1 Asterisk does not meet assumptions of chi-square test.

Associations with questions regarding barriers to the shift to a more plant-based diet and age were also found (Table 4). Regarding personal barriers to making the shift, “lack of interest” was more frequently associated with older respondents (*P* = 0.013). With respect to barriers for clients, citing “perceived difficulty” was more frequent among younger respondents (*P* = 0.006) while “other” was more frequent among older respondents. An association was also found with DPG membership and client barriers, with DPG nonmembers more frequently associated with the “other” response (*P* = 0.047).

**Table 4:** Associations between age and DPG membership and responses to barrier questions

Question	Frequency answering YES	Frequency answering YES	*P*-value^1^
What are some barriers to you implementing this recommendation for yourself?^2^	Age group <42 years	Age group >42 years	
Lack of interest in consuming plant proteins	7	14	0.013
Perceived difficulty in following a plant-based diet	6	6	ns
Lack of knowledge about the quality of plant proteins	4	3	*
Lack of knowledge about the health benefits of consumingplant proteins	1	4	*
Other	7	3	ns
Reflecting on the clients that you work with, what are some barriers to implementing this recommendation?^2^	Age group <42 years	Age group >42 years	
Lack of interest in consuming plant proteins	47	44	ns
Perceived difficulty in following a plant-based diet	48	31	0.006
Lack of knowledge about the quality of plant proteins	35	28	ns
Lack of knowledge about the health benefits of consumingplant proteins	28	23	ns
Other	9	19	0.027
Reflecting on the clients that you work with, what are some barriers to implementing this recommendation?^2^	DPG member	DPG nonmember	
Lack of interest in consuming plant proteins	46	47	ns
Perceived difficulty in following a plant-based diet	39	40	ns
Lack of knowledge about the quality of plant proteins	29	33	ns
Lack of knowledge about the health benefits of consuming plant proteins	25	25	ns
Other	8	20	0.047

1 Asterisk does not meet assumptions of chi-square test.

2 Recommendation for shift to more plant-based diet.

Additional associations were found between respondent age and where information on protein quality is obtained (Table 5). Younger respondents were more likely to indicate college training (*P* = 0.001) while older respondents were more likely to indicate using professional meetings and conferences (*P* < 0.0001) and continuing education classes (*P* = 0.041) as sources for learning more about plant protein quality.

**Table 5:** Associations between age and sources of information on protein quality

Question	Frequency answering YES	Frequency answering YES	*P*-value^1^
Where do you get information on protein quality of dietary proteins?^2^	Age group <42 years	Age group >42 years	
College training	49	29	0.001
ADA communication	35	32	ns
Government agencies (CDC, USDA, FDA, HHS, etc.)	30	29	ns
Professional meetings and conferences	18	39	<0.0001
Scientific journals	31	31	ns
Continuing education courses	13	23	0.041
Internet	26	31	ns
I do not look for information on protein quality	9	6	ns
Other	1	2	*

1 Asterisk does not meet assumptions of chi-square test.

2 More than one answer permitted.

## Discussion

A meta-analysis of 56 online surveys (Cook et al. 2000) reported a response rate of 35% while a review by Sheehan (2012) found that response rates for online surveys have declined from 62% in 1986 to 24% in 2000, concluding that the year the survey was done and the number of follow-up contacts most influenced response rates. For this survey, only one notification was sent, resulting in a 10% response rate. Follow-up emails may have improved the response rate.

Since the survey was exploratory in nature, the beliefs and knowledge among dietitians about protein quality in plant-based diets were not predicted in advance. Responses to belief statements were generally supportive of plant-based diets as indicated by greater than 75% of respondents expressing disagreement with the statement that only animal proteins are high quality and nearly two-thirds of respondents disagreeing that all plant proteins are incomplete. There was very high agreement (93%) with the concept of complementary proteins (combining plant proteins to form complete proteins), indicating this is a widely accepted concept. In line with these beliefs, nearly two-thirds of respondents did not express concern with recommending a plant-based diet. Likewise, two-thirds of respondents said that they take protein quality into account when making diet recommendations. However, less than 50% indicated that an understanding of protein quality was important to their practice. It may be that while recognition of protein quality in a general sense is considered important, specific knowledge of protein quality determination methods is not a priority.

Responses to the knowledge questions indicated that the understanding of protein quality of plant proteins in general is lacking among a fairly high percentage of respondents. While 97% of the respondents correctly identified wheat and rice as incomplete proteins, 25% of the respondents incorrectly identified peanut protein as complete and 27% were not able to identify soy protein as a complete protein. A general question about the factors that are included in protein quality determination found that 40% of the respondents did not identify the amino acid needs of humans and 36% did not identify the digestibility of the protein as being important criteria. Even greater knowledge gaps were apparent with questions about specific protein quality methods of measurement. The only protein quality measurement that scored high for familiarity was BV. It is possible that this was more indicative of a familiarity with the general usage of the term BV versus familiarity with the specific BV method. While the PDCAAS method is the most widely used method for protein quality determination in the United States and globally, only 18% of the respondents were familiar with this method, only 10% correctly identified it as the method used by the FDA for nutrition labeling, and only 11% correctly identified it as the method cited by AND in their position paper on vegetarian diets. Furthermore, a fairly high percentage of respondents did not identify two of the underlying tenets of the method as being important to protein quality determination: amino acid needs of humans (40%) and digestibility of a food protein (36%). This suggests that more could be done to increase dietitians' understanding of protein quality measurement. Since 57% of all respondents indicated that they get their knowledge of protein quality from their college training and 49% from AND communications, these avenues would be potential ways to incorporate this training. Professional meetings and conferences might be more effective in reaching older practitioners.

The barrier to moving to a more plant-based diet that was cited most frequently, both for the dietitians personally and for their clients, was lack of interest. While it is beyond the scope of this study, an exploration of the reasons for this apparent lack of interest would be of great interest for further research.

An objective of the study was to determine if beliefs and knowledge of protein quality of plant-based diets would differ by demographics such as DPG membership and practice setting. No associations were found with any of the belief questions. Since both individual DPG membership and practice settings were quite diverse, the numbers of respondents in any particular category were quite small, making it difficult to detect differences. However, DPG membership overall (member vs. nonmember) was significantly associated with the knowledge question about familiarity with test methods, specifically with the AAS method. DPG membership may be a surrogate marker for a more engaged practitioner. Associations with this question and age were also found. Younger respondents were more likely to indicate familiarity with the BV method. As stated previously, this may not represent true familiarity with the method, but rather the more general concept, but it may be indicative that this terminology is more frequently used in today's curricula. The association found between older respondents and not being familiar with any methods of protein quality determination may again be an indication that more attention is now being paid to the concept of protein quality but based on responses to the knowledge questions, training could be improved.

Associations between age and where information on protein quality is obtained are likely a reflection of the time elapsed since graduation, with a higher frequency of younger respondents indicating they get this information from their college training while older respondents were more likely to indicate professional meetings and conferences and continuing education classes as sources of information.

A limitation of this study is that although the sample group tracked well with the demographic characteristics of the study population, it may not be generalizable to the larger population of dietitians. In particular, the low level of vegetarian DPG membership of both the study sample and population may be a factor in the finding of lack of knowledge about plant-based proteins. Additionally, a larger sample size would have been useful in detecting differences between practice groups. However, as a first attempt at assessing the current state of beliefs and knowledge among dietitians regarding plant-based diets, the results of this study will provide a baseline for further study and may spur discussion about education in the area of protein quality in general. Further study is also warranted to better understand barriers in shifting to a more plant-based diet.

## Conclusions

The findings of this study suggest that dietitians are generally supportive of plant-based diets but their knowledge of plant-based protein quality in general, and methods of protein quality measurement more specifically, could be enhanced in order to provide them with a better knowledge base for making dietary protein recommendations. Incorporating additional training in this area via university curricula and continuing education opportunities for practitioners, along with professional meetings and conferences, may be appropriate, although additional research is needed to better characterize the need.
